# Evaluation of the Interstitial Histological Lesions in Pulmonary Langerhans Cell Histiocytosis*

**DOI:** 10.5146/tjpath.2022.01591

**Published:** 2023-09-15

**Authors:** Halide Nur Urer, Hatice Dincer

**Affiliations:** Department of Pathology, University of Health Sciences Turkey, Haseki Training and Research Hospital, Istanbul, Turkey; University of Health Sciences Turkey, Istanbul Training and Research Hospital, Istanbul, Turkey

**Keywords:** Histiocytosis, Langerhans-Cell, Pulmonary fibrosis, Smoking

## Abstract

***Objective:*** Pulmonary Langerhans cell histiocytosis is a cystic lung disease characterized by the proliferation of parenchymal dendritic cells. The disease can become chronic or even cause pulmonary fibrosis. Our aim in this study was to investigate the typical histological findings and interstitial fibrosis in pulmonary Langerhans cell histiocytosis cases.

***Material and Methods:*** In the study, cases that had undergone diagnostic resection were screened. Smoking, histological stage (subacute, subacute-chronic), and cystic and eosinophilic granulomas were confirmed in the cases. In addition to emphysema, chronic nonspecific bronchiolitis, interstitial fibrosis (subpleural-paraseptal fibrosis, peribronchial fibrosis, fibrotic nonspecific interstitial pneumonia), honeycomb-type fibrocysts, and unexpected lesions were investigated. Descriptive and comparative (Fisher exact test) statistical analyses were used in the study (p<0.05).

***Results:*** A total of 27 cases were detected; age distribution was 17-68 (36.4). Smoking was present in 15 (55.5%) cases. Six (22.2%) cases were subacute, and 21 (7.7%) cases were subacute-chronic histological stage. A cystic lesion was present in 22 (81.4%) cases. All cases had emphysema accompanying the underlying lesions. Chronic nonspecific bronchiolitis was detected in 14 (51.8%) cases. Interstitial fibrosis was detected in 8 (29.6%) patients. Compared to interstitial fibrosis and nonfibrosis, there was no significant difference between being younger than 39 years, gender, smoking, and histological stage (p=0.41; 1; 0.69; 0.63, respectively).

***Conclusion:*** There is a risk of developing interstitial fibrosis patterns and honeycomb-type fibrocysts in the progression of pulmonary Langerhans cell histiocytosis. Histopathological evaluation can play an important role in the detection of risk groups.

## INTRODUCTION

Pulmonary Langerhans cell histiocytosis (LCH) is a cystic lung disease characterized by the proliferation of parenchymal dendritic cells. A significant portion of adult cases are related to smoking and only the lung is involved; others have systemic disease (1–4).

The typical pathological finding is nodular eosinophilic granulomas composed of bronchiolocentrally located Langerhans cells and eosinophils (5). In the following period, centriacinar emphysema develops with airway destruction and fibroblastic activity, and collagenous composition increases while inflammation decreases. As a result, stellate-shaped scars and fibrosis develops around the cystic spaces. Pulmonary hypertension and interstitial fibrosis may develop in chronic cases (6,7).

In the tissues of surgical resection, it is possible to diagnose the disease and determine the fibrotic stage. The disease that becomes chronic has a risk of resulting in interstitial fibrosis. Our aim in this study was to investigate the characteristics of histological lesions in pulmonary LCH and their relationship with interstitial fibrosis.

## MATERIAL and METHOD

The study screened pulmonary LCH cases between the ages of 17-80 who had undergone surgical resection between 2003-2021 and reported in the Department of Pathology. Cases whose preparations were not available or who were diagnosed with limited biopsy were excluded. Archival slides of the cases were re-examined. Immunohistochemical analysis confirmed the accuracy of the diagnosis. Demographic data and smoking history were recorded from the case files.

Interstitial, peribronchial eosinophilic granulomas were accepted as the diagnostic criteria. In the examination, lesions were grouped as sub-acute and subacute-chronic. Granulomas consisting of abundant eosinophils without fibrosis were considered as subacute. Peribronchiolar fibroblastic proliferation forming stellate-shaped scar nodules were described as subacute-chronic stage lesions. Emphysema, chronic nonspecific bronchiolitis, interstitial fibrosis (subpleural-paraseptal fibrosis, peribronchial fibrosis, fibrotic nonspecific interstitial pneumonia), honeycomb-type fibrocysts, and atypical lesions accompanying all these lesions were investigated.

LCH cases with interstitial fibrosis were compared with the others in the group according to age, gender, smoking, and histological stage.

The study design has been approved by the local ethics committee (University of Health Sciences Yedikule Chest and Thoracic Surgery Research and Training Hospital Ethics Committee). The study protocol number/date was 2022-186/13.01.2022.

In the study, descriptive statistical calculations were performed regarding the distribution of demographic and histological lesions. Fisher exact test was used to compare the characteristics of patients with interstitial fibrosis (p<0.05).

## RESULTS

A total of 29 pulmonary LCH cases were detected. Two cases were excluded from the study due to the inaccessibility of the preparations and the fact that the diagnosis had been made with a transbronchial biopsy. Histopathological evaluation of the remaining 27 cases was performed. Demographic and essential pathological findings of the cases are summarized in Table 1. Diagnoses were confirmed by S-100 and/or CD1a immunohistochemical expression.

**Table 1:** General characteristics of the cases.

**Features**	**n=27 (%)**
Age	17-68
Mean±SD	36.4± 13.11
Gender	
Woman	10 (37)
Male	17 (62.9)
Smoking	
Yes	15 (55.5)
No	12 (44.4)
Stage of the disease	
Subacute	6 (22.2)
Subacute-Chronic	21 (77.7)
Eosinophilic granulomas	
Yes	27 (100)
No	0 (0)
Cystic lesions	
Yes	22 (81.4)
No	5 (18.5)

**Figure 1:**
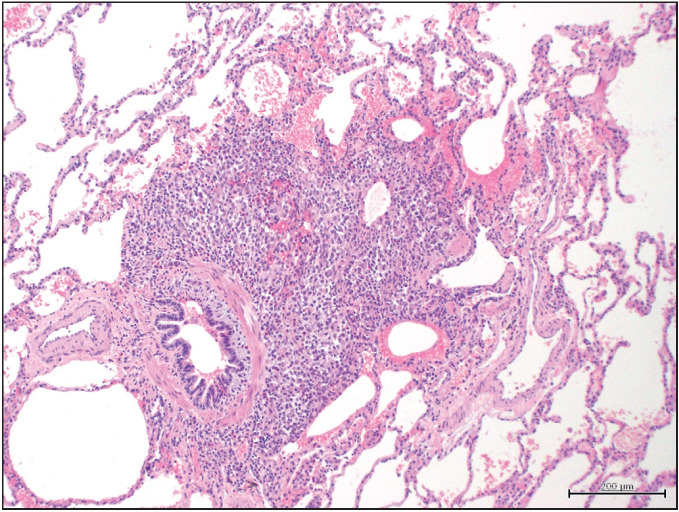
Peribronchial granuloma, H&Ex200.

**Figure 2:**
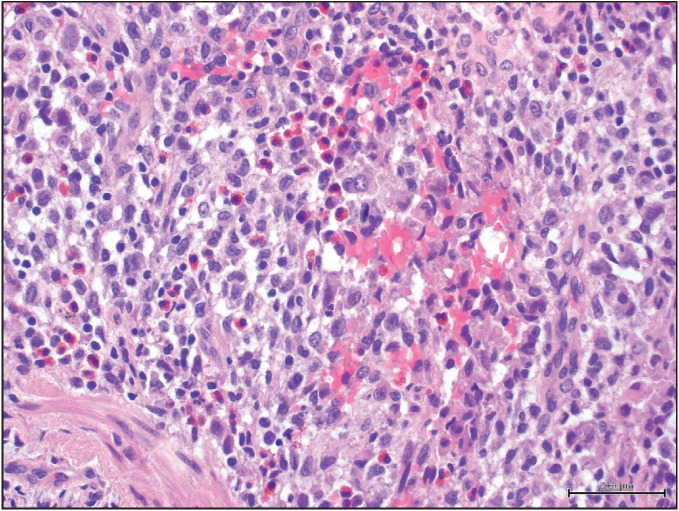
Langerhans cells and eosinophils, H&Ex400.

Peribronchial eosinophilic granulomas were detected in all cases (Figure 1). Nodular granulomas contained varying proportions of Langerhans cells and eosinophils (Figure 2). Emphysema was present in all cases. Chronic nonspecific bronchiolitis was detected in 14 (51.8%) cases.

**Figure 3:**
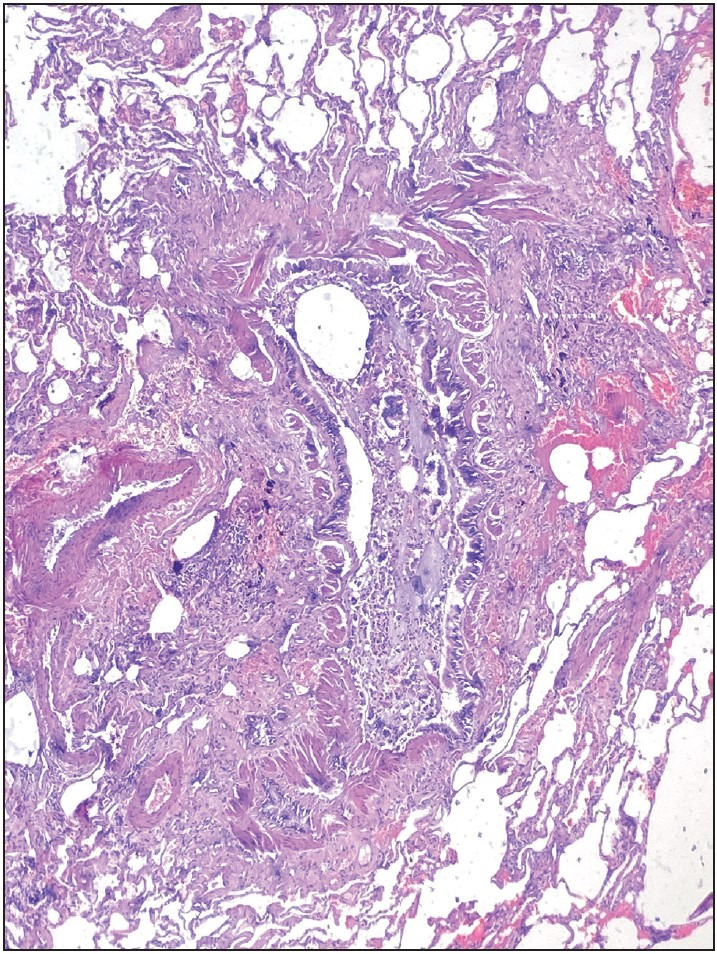
Peribronchial fibrosis, H&Ex100.

**Figure 4:**
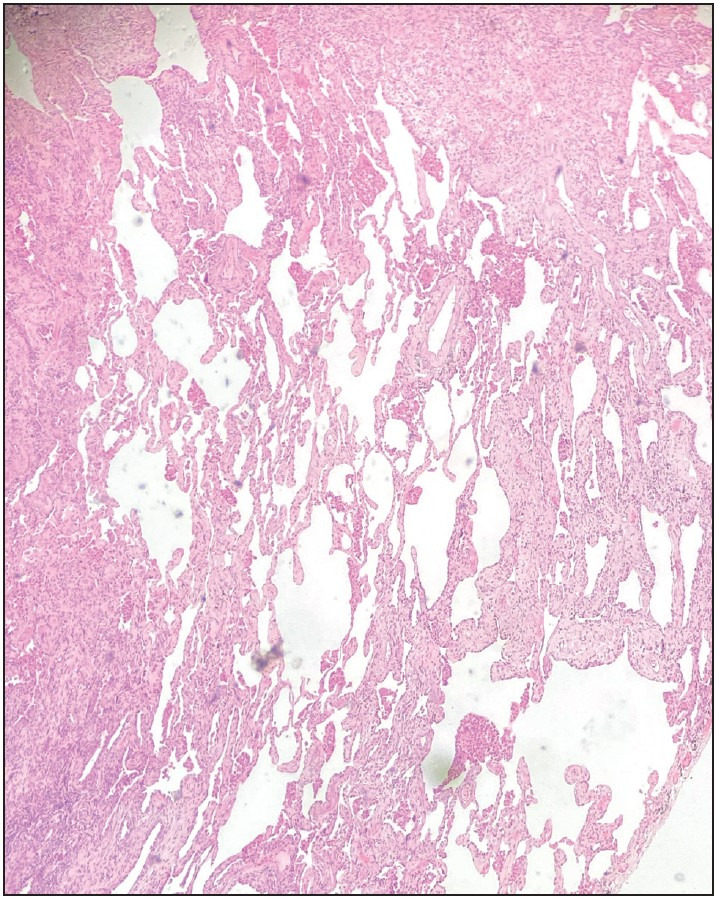
Fibrotic nonspecific interstitial pneumonia, H&Ex200.

**Figure 5:**
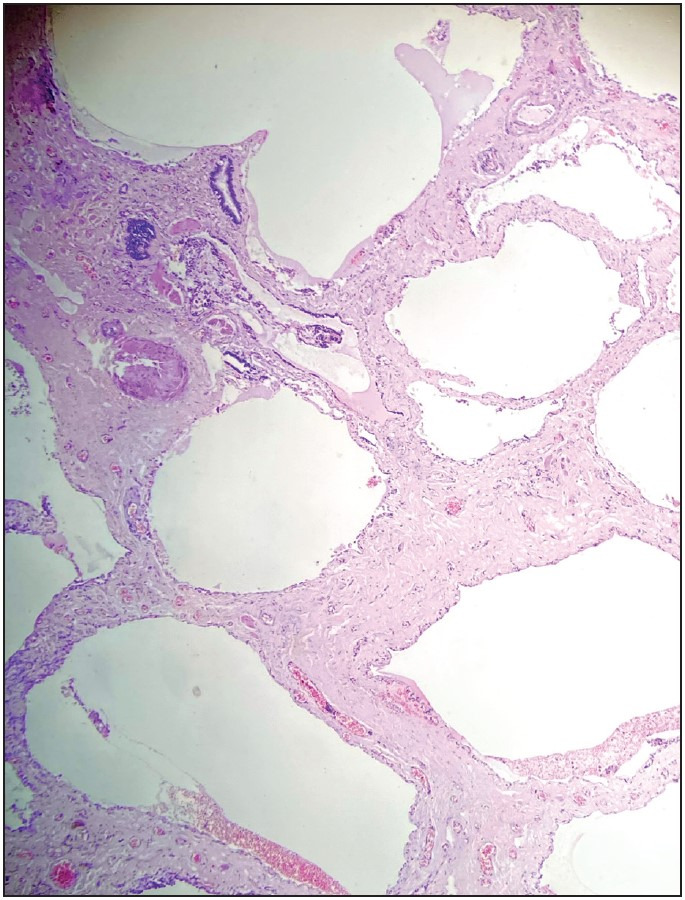
Honeycomb fibrocysts, H&Ex200.

Interstitial fibrosis was detected in 8 (29.6%) patients. The histologic pattern of the interstitial fibrosis was diverse. Peribronchial fibrosis, fibrotic type nonspecific interstitial pneumonia, and subpleural and paraseptal fibrosis patterns were observed (Figure 3, Figure 4, Figure 5). The gender, smoking, and histological stage findings in cases with interstitial fibrosis patterns are shown in Table 2.

**Table 2:** Features of the interstitial fibrotic patterns.

**Fibrosis patterns**	**n (%)**	**Gender** **Female/Male**	**Smoking** **Yes/No**	**Subacute/** **subacute-chronic**
Peribronchial fibrosis	3/27 (11.1)	0/3	3/0	1/2
Fibrotic nonspecific interstitial pneumonia	3/27 (11.1)	2/1	2/1	0/3
Subpleural, paraseptal fibrosis	2/27 (7.4)	1/1	0/2	0/2

**Figure 6:**
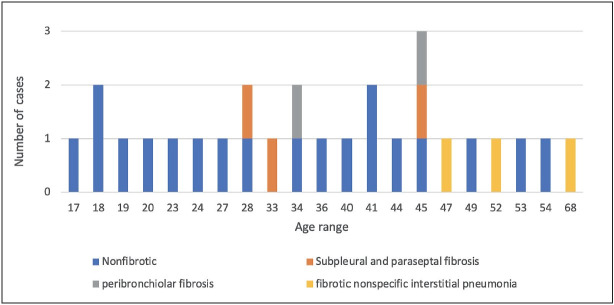
Distribution of interstitial fibrosis according to age.

The age range of the eight patients with interstitial fibrosis patterns was 28-68 years (Figure 6).

When LCH with interstitial fibrosis was compared with nonfibrotic cases, there was no significant difference regarding being younger than 39 years of age, gender, smoking, or histological stage. (p=0.41; 1; 0.69; 0.63, respectively) (Table 3).

**Table 3:** P value in LCH with and without interstitial fibrosis.

**Features**		**LCH with interstitial fibrosis (n=8)** **n (%)**	**LCH without interstitial fibrosis (n=19)** **n (%)**	**p value**
Age	<39	3 (37.5)	11 (57.8)	0.41
	>39	5 (62.5)	8 (42.1)	
Gender	Female	3 (37.5)	7 (36.8)	1
	Male	5 (62.5)	12 (63.1)	
Smoking	Yes	5 (62.5)	10 (52.6)	0.69
	No	3 (37.5)	9 (47.3)	
Histological stage	Subacute	1 (12.5)	5 (26.3)	0.63
	Subacute-chronic	7 (87.5)	14 (73.6)	

Other features of the histological evaluation of the cases are shown in Table 4. Two cases with honeycomb fibrocysts were detected. One of these cases had fibrotic nonspecific interstitial pneumonia and the other had peribronchial fibrosis.

**Table 4:** Other histopathogical lesions.

**Features**	**n (%)**
Honeycomb cysts	2/27 (7.4)
Bronchiolization	1/27 (3.7)
Organized pneumonia	1/27 (3.7)
Smoking-related respiratory bronchiolitis	1/27 (3.7)
Atypical alveolar hyperplasia	1/27 (3.7)
Pleural talcosis	1/27 (3.7)

## DISCUSSION

In our study, interstitial fibrosis was found in 29.6% of subacute and subacute-chronic LCH cases. LCH often presents with a stable clinical course with spontaneous regression or smoking cessation. Some patients develop recurrence, pulmonary hypertension, and progression (7). Lesions accompanying essential histology may be helpful in predicting the course of the disease.

More than half of all our cases and 62.5% of the cases with interstitial fibrosis were smokers. It is reported that smoking causes BRAF signal pathway activity and mutation in the cell (7–9). In the study of Kamionek et al., 30% *BRAF, KRAS G12C*, and *MAP2K1* alterations are found in smokers pulmonary LCH (10). The *BRAF V600E* mutation that develops on this pathway leads to cellular aging (11). Cellular senescence can lead to the development of fibrosis in pulmonary LCH. Therefore, categorizing pulmonary LCH according to smoking seems to be a more accurate approach.

Pulmonary LCH creates of the disease complex with smoking-associated respiratory bronchiolitis and desquamative pneumonia (12). That is why it is usual for it to accompany eosinophilic granulomas (13). Eosinophils and Langerhans cell destructive granulomas cause respiratory bronchiolitis and centriacinar emphysema (14). In the clinical features, the development of spontaneous pneumothorax is a typical finding of the disease (15–17). Fibrotic emphysema develops in the progression of long-lasting disease (18). The presence of emphysema in the subacute-chronic stages in our cases may be a sign that the destructive effect develops early.

It has been suggested that fatal end-stage fibrosis may develop in pulmonary LCH (19). Honeycomb cysts, old age, and the obstructive type of respiratory dysfunction are among the negative factors (10,11,13). Also, histology may be accompanied by fibrosis along the alveoli in the interstitium surrounding the severely inflamed nodules (5,11). In our study, honeycomb fibrocysts as well as interstitial fibrosis patterns were detected. Peribronchial fibrosis is common in lesions that become chronic due to the peribronchial location of eosinophilic granulomas (20). Fibrotic nonspecific interstitial pneumonia and subpleural/paraseptal fibrosis may be subtypes of rare progressive fibrosis. As a result, chronic pulmonary LCH may occur in a heterogeneous pattern.

In our study, we encountered accompanying rare, organized pneumonia, bronchiolization, atypical alveolar hyperplasia, and pleural talcosis. It is emphasized that some of these, such as organized pneumonia, may develop secondary to the disease (13). Pleural talcosis is the iatrogenic result of pneumothorax treatment.

There are some limitations in our study. Since the first of these cases were not followed up in a single center, sufficient information about their prognosis could not be obtained. For the same reason, clinical and radiological data could not be accessed.

## CONCLUSION

The histopathological features of pulmonary LCH, which is rare, may show unexpected diversity. In the advanced stage of the disease, there is a risk of developing interstitial fibrosis patterns and honeycomb-type fibrocysts. Histopathological evaluation helps to identify risk groups. Close follow-up of patients with pulmonary LCH who are candidates for interstitial fibrosis are recommended.

## Conflict of Interest

As the authors of our manuscript, we declare that there is no conflict of interest regarding the publication of this paper.
